# Factors associated with timely HIV testing of sexual partners identified through partner services in lower eastern Kenya, 2023–2024

**DOI:** 10.1371/journal.pgph.0007171

**Published:** 2026-11-13

**Authors:** Jones Mutiso, Patrick Eshikumo, Christian Onyango, Felix Makori, John Kioko, Virginia Karanja, Agnes Langat, Jonathan Mwangi, Peter Kyalo, Caroline Ngeno, Emily Koech, Sam Wafula

**Affiliations:** 1 Center for International Health Education and Biosecurity (CIHEB), Nairobi, Kenya; 2 Division of Global HIV & TB (DGHT), Global Health Center (GHC), US Centers for Disease Control & Prevention (CDC), Nairobi, Kenya; University of Health and Allied Sciences, GHANA

## Abstract

Partner Services (PS) is a key strategy for closing HIV case identification gaps by facilitating testing among sexual partners of people living with HIV (PLHIV). Timely testing is essential for early diagnosis, prompt linkage to care, and interruption of HIV transmission, yet evidence on factors influencing timely partner testing in resource-limited settings is limited. We analyzed routinely collected data from 2,210 index clients between January 2023 and December 2024. we defined timely testing as HIV testing within 14 days of partner elicitation, and examined associated factors using generalized linear mixed models. Females comprised 63% of index clients while males accounted for 58% Provider referral was the most common notification approach (63%), whereas contract referral was least used (8%). Compared with other approaches, dual referrals (aOR 2.22, 95% CI 1.34-3.66) and provider referrals (aOR 1.76, 95% CI 1.13-2.76) were significantly associated with timely testing. Timely testing was also more likely among index clients over 50 years, those who were unemployed or self-employed, and those identified through social network strategy. Conversely, partners of single index clients, and those linked to secondary index clients were less likely to test within 14 days. Among the sexual partners, formal employment, and HIV positive results were associated with timely testing. These findings suggest that PS delivery approaches and client characteristics influence timely partner testing and strengthening provider and dual referrals while targeting younger, single, and secondary index partners could improve timely identification of PLHIV.

## Introduction

Globally, an estimated 14% of people living with HIV (PLHIV) are unaware of their HIV status [1]. In Kenya, significant progress has been made towards closing this gap, with 96% of PLHIV being aware of their status, surpassing the first 95% of the UNAIDS 95-95-95 target for awareness of HIV status amongst PLHIV. According to the 2023 estimates from the National Syndemic Diseases Control Council (NSDCC), about 70,000 PLHIV were unaware of their HIV status, highlighting the need for targeted last mile efforts to close the gap. The scale-up of partner service (PS) in Kenya in 2017 in response to the 2016 WHO guidelines [2] has been instrumental in Kenya’s progress towards improved identification and linkage to care for PLHIV. As the country progresses towards achieving epidemic control, innovative strategies such as PS remain essential.

Partner Service is a voluntary process in which individuals diagnosed with HIV (index clients) are assisted by trained healthcare providers to identify and notify their sexual and/or drug injecting partner(s) about potential exposure to HIV infection with the goal of offering them HIV testing services (HTS) [3,4]. In Kenya, PS is implemented using a structured process as shown in Fig 1, “ Partner Services Algorithm*”*, and is broadly categorized into passive and assisted approaches. In passive PS, index clients are encouraged to notify their sexual partners and refer them for HIV testing (also known as client referral). In contrast, assisted PS (aPS) involves active provider support and includes three main approaches, including 1). Dual referral where an index client works with the HTS provider to disclose their HIV status and have the sexual partner tested, 2). Contract referral where index clients commit to notify their sexual partners and have them tested within 14 days beyond which the HTS provider notifies the sexual partner(s), and 3). Provider referral where the HTS provider contacts the sexual partners directly and offers them HIV testing [3,4]. The index client makes an informed choice about the notification approach to be used to reach their sexual partner(s), upon guidance by the HCW about available sexual partner notification approaches, which is eventually used to follow up the named sexual partner(s).Timely HIV testing of sexual partners is critical for identifying undiagnosed HIV infections, linking people living with HIV to treatment, and providing HIV prevention services to people without HIV, all of which breaks the chain of HIV transmission and advances epidemic control. Evidence shows that effectiveness and timeliness of PS outcomes vary by approach [3–5], with the Kenya HIVST and aPNS operational guidelines defining timely HIV testing as occurring within 14 days of elicitation. Furthermore, the characteristics of both index clients and their partners may influence the timeliness of partner testing.

**Fig 1:**
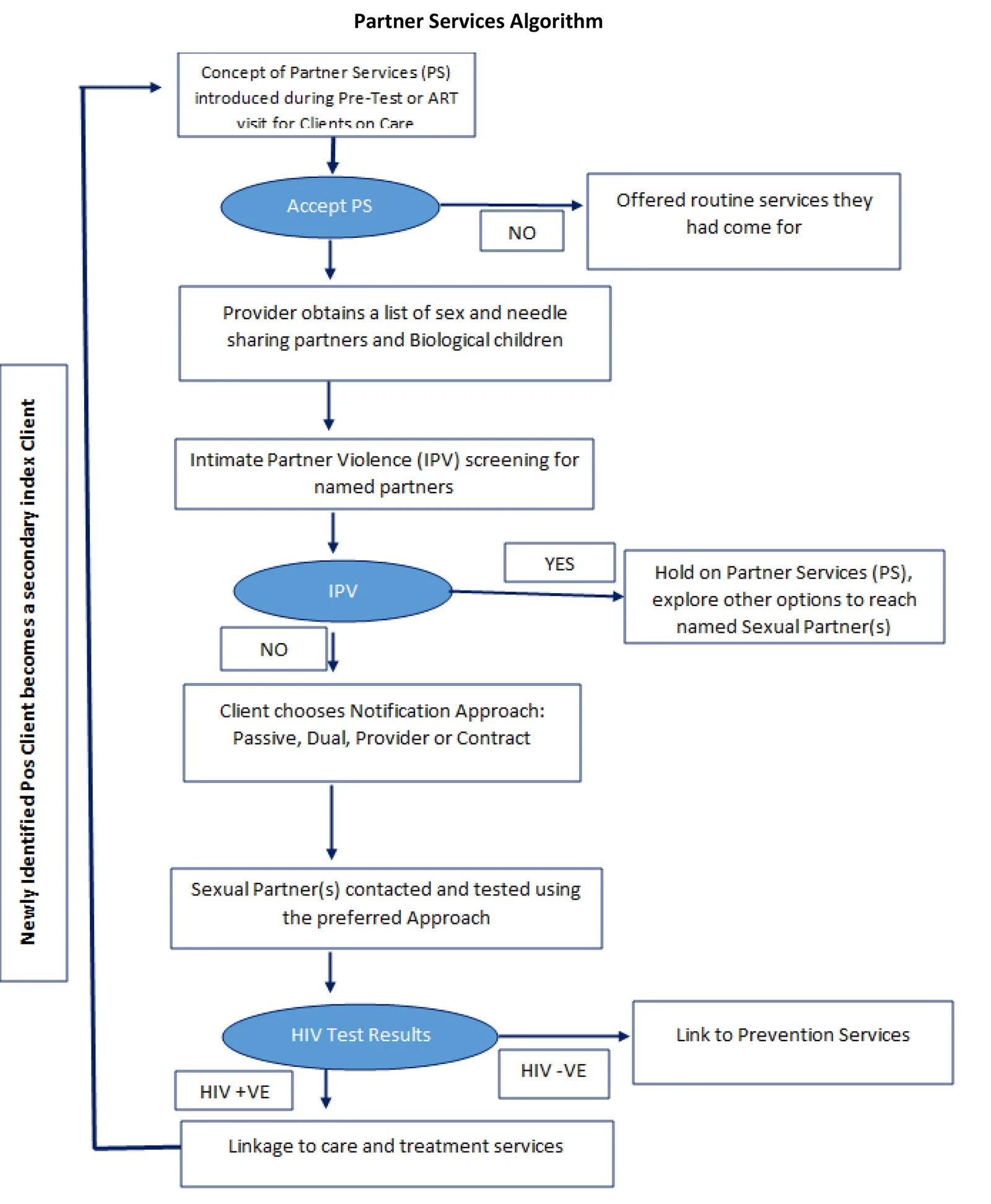
Partner Services Algorithm.

Identifying the most time-efficient PS approach is particularly important in resource-constrained settings like Kenya, where optimizing use of limited resources is critical. However, local data on the relative efficiency of these approaches remains limited. This evaluation sought to address this gap by analyzing programmatic data to determine factors associated with testing within 14 days, including socio-demographic characteristics of the index clients and their sexual partner(s). These insights can inform strategies to enhance HIV case identification, particularly among priority populations such as young women aged 15–24 years and other populations at elevated risk, who are disproportionately affected by HIV and lag in identification of those living with HIV.

## Materials and methods

### Ethics statement

This study was approved by the Ethics and Scientific Review Committee at AMREF under the approval number AMREF-ESRC P1421/23, and the University of Maryland, Baltimore Institutional Review Board under approval number HP-00106405. This evaluation was also reviewed by U.S. CDC human research protection procedures and was determined to be non-research. While the data for this study was abstracted from the Kenya electronic medical records, all our participants provided informed consent for participation in partner services at partner elicitation and testing. Further, all authors provided individual concurrence statements to affirm their consent to publish this study.

### Study population

The study population was PLHIV above 15 years who had consented to receive partners services (index clients), either newly diagnosed with HIV or already on HIV care [4], and their sexual partners who had received a test. According to the national PS guidelines, eligible partners include both sexual and drug-injecting partners, however, this study was limited to sexual partners excluding drug-injecting partners due to their very low numbers and the study's focus on sexual partner notification outcomes.

The index clients were either primary (i.e., initial person who tests positive for HIV then provides names of sexual partners they wish to be traced for HIV testing as well.) or secondary (i.e., these are sexual partners of the primary index client who are traced, tested and subsequently diagnosed with HIV. They are also enrolled in partner services, as secondary index clients, to elicit more sexual partners). The index clients included both newly diagnosed and those already on care including the high viral load clients (HVL), return-to-care (RTC), HIV infected mothers receiving prevention of mother to child transmission (PMTCT) services, among others. The sexual partners were elicited during post-test counseling for the newly diagnosed HIV-positive clients and during follow up clinic appointments for clients in care, and reached through passive, dual, contract or provider notification approaches. Sexual partners elicited from these index clients were included if they had received an HIV test by 30 June 2025.

### Study design and setting

A retrospective cohort analysis was conducted between January 2023 and December 2024, using HIV index testing data from 115 Ministry of Health (MOH) owned public health facilities providing HTS and supported by Center for International Health Education and Biosecurity (CIHEB)-Kenya in the Kitui, Machakos, and Makueni Counties.

### Data collection and management

Data were abstracted from Kenya Electronic Medical Records (EMR) between 1^st^ and 24^th^ March 2025, using a script that covered all the variables of interest. Data was de-identified, encrypted and protected using access passwords. Data cleaning and pre-processing were performed to ensure accuracy, consistency, and completeness before analysis using tidyverse package [6]. Exploratory data analysis was conducted to identify anomalies, patterns, and outliers using statistical summaries and visualizations.

The primary outcome for this study was time taken in days from elicitation of sexual partners to the time they were tested for HIV, with this study considering timeliness as sexual partners being tested within 14 days. Independent variables included index testing notification approach, age of index client and sexual contact, sex, occupation, marital status, relationship of listed sexual contact with the index client, sexual contact’s HIV status during line listing, population type (epidemiologically at high-risk population vs general population) and HIV status of the tested sexual contact.

### Data analysis

We used R version 4.5.0 for statistical analysis. A generalized linear mixed model was fitted using maximum likelihood estimation with Adaptive Gauss-Hermite Quadrature to generate odds ratios (ORs) at 95% confidence intervals (CIs). The model employed a binomial family with a logit link function to analyze the binary outcome of sexual contacts receiving a test within 14 days. The fixed effects included age group, occupation of both index and sexual contacts, enrollment into care, marital status, partner services approach, index client type, HTS modality, type of index client, and sexual contacts’ prior knowledge of HIV status. Index client characteristics were incorporated to correct for non-independence within individual observations.

A multivariable generalized linear mixed-effects model (GLMM) with a logit link and binomial distribution was fitted to estimate adjusted odds ratios (aORs) and 95% confidence intervals for timely HIV testing (defined as testing within 14 days of sexual partner elicitation). A mixed-effect approach using a GLMM was chosen over conventional multivariable logistic regression to account for non-independent and hierarchical structure of the data. Clustering of multiple sexual partners within the same index clients violates the assumption of independent observations required for conventional logistic regression. A random intercept for index client was included to account for clustering of multiple sexual partners within the same index client. Covariates included in the multivariable model were selected based on epidemiological relevance, published literature, and biological plausibility. Variables adjusted for included index client age, occupation, marital status, HIV care enrollment status, Client entry point, index client type, population type, partner notification approach, sexual partner age, occupation, and prior knowledge of HIV status. These variables were included simultaneously to minimize potential confounding.

Though not shown, a rigorous model assumption checks were conducted to ascertain the validity and reliability of the statistical inference drawn from the model. The results of post estimation analysis, posterior predictive check including Akaike Information Criterion (AIC), corrected AIC, conditional R², Root Mean Square Error (RMSE), and Intra-class Correlation Coefficient (ICC) indicated reasonable model fit.

## Results

### Sociodemographic characteristics of the respondents

Table 1 shows that among the 2,210 index clients, females were the majority (n = 1393; 63%) and the median age was 37 years. Majority of the index clients were aged 35–49 years (n = 909; 41%), were self-employed (n = 1280; 58%), were married in a monogamous relationship (n = 1,214; 55%) and elicited one contact (n = 1668; 78%). Further, most of the index clients were enrolled for HIV care (n = 2013; 91%), were primary index clients (n = 1256; 57%), were from the general population (n = 2022; 91%) and were tested for HIV through the provider-initiated testing and counseling (PITC) hospital testing modality where clients were referred for testing by health care worker based on HIV risk (n = 1294; 59%).

**Table 1:** Characteristics of index clients’ and their tested sexual partners, lower eastern Kenya.

Characteristic	Index N = 2210	Sexual Partners N = 2,906
**Median (IQR) Age in Years**	37 (29, 46)	36 (28, 45)
**Sex**		
Male	817 (37%)	1,679 (58%)
Female	1,393 (63%)	1,227 (42%)
**Age group**		
15-24	233 (11%)	470 (16%)
25-34	657 (30%)	862 (30%)
35-49	909 (41%)	1,118 (38%)
50+	411 (19%)	456 (16%)
**Occupation**		
Self employed	1,280 (58%)	1,387 (48%)
Not employed	310 (14%)	671 (23%)
Formal Employment	256 (12%)	334 (11%)
Informal Employment	364 (16%)	514 (18%)
**Marital status**		
Married Monogamy	1,214 (55%)	
Widowed	107 (4.8%)	
Separated/ Divorced	271 (12%)	
Married Polygamy	64 (2.9%)	
Single	554 (25%)	
**# of Contacts elicited**		
Elicited 1	1,668 (75%)	
2-3 elicited	505 (23%)	
4 + elicited	37 (1.7%)	
**Enrolled to HIV care**		
Enrolled	2,013 (91%)	
Unknown	197 (8.9%)	
**Index client type**		
Secondary	954 (43%)	
Primary	1,256 (57%)	
**Population type**		
General Population	2,022 (91%)	
Epidemiologically at-risk Populations	188 (8.5%)	
**HTS modality**		
Community Testing	35 (1.6%)	
PITC Hospital Patient Testing	1294 (59%)	
Index testing	572 (26%)	
Social Networks	161 (7.3%)	
PITC non-patients	148 (6.7%)	
**HTS Notification Approach**		
Passive Referral		374 (13%)
Contract Referral		237 (8%)
Dual Referral		464 (16%)
Provider Referral		1,831 (63%)
**Median days between elicitation and receiving HIV test (IQR)**		43 (4, 179)
**HIV test outcome**		
Negative		2,370 (82%)
Positive		536 (18%)
**Days between elicitation to receiving HIV test by category (in days)**		
<15		994 (34%)
15-29		283 (9.7%)
30-59		319 (10.9%)
60+		1,310 (45%)
**Linked to ART**		
Linked to ART		427 (80%)
Not linked to ART		109 (20%)
(HIV Negative)		2,370

Abbreviations: IQR = Interquartile Range, HIV = human immunodeficiency virus, PS = partner services, HTS = HIV Testing Services, PITC = Provider-initiated testing and counseling, ART = Antiretroviral Therapy.

Of the 3,938 sexual partners elicited, 2,906 (74%) were tested with majority being male (n = 1679; 58%). The median age of the sexual partners elicited was 36 years with most within the 35–49-year age bracket (n = 1118, 38%). Most were self-employed (n = 1387; 48%), HIV negative (n = 2370; 82%) and their preferred HTS notification approach was the provider referral (n = 1831; 63%). Only 34% (n = 994) of the sexual partners that were elicited received timely testing within 14 days with a median duration between elicitation and HIV testing being 43 days.

### A multivariable analysis of the factors associated with HIV testing of sexual contacts

Table 2 presents factors associated with timely HIV testing among sexual partners. Compared with partners aged 15–24-years, sexual partners aged 50 years and higher odds of timely HIV in the unadjusted analysis (OR=1.60; 95% CI: 1.18-2.16). However, this association was no longer statistically significant after adjusting for other factors in the model. Similarly, compared with sexual partners of index clients aged 15–24 years, sexual partners of index clients aged 50 years and above had higher odds of timely HIV testing in the unadjusted analysis (OR=1.40; 95% CI: 1.00-1.95). After adjustment for potential confounders, sexual partners of index clients aged 50 years and above had 3.8 increased odds of timely HIV testing than sexual partners of index clients aged 15–24 years (aOR=3.84; 95% CI: 1.35-10.90).

**Table 2:** Factors associated with timely testing (≤ 14 days) in partner services, lower eastern Kenya.

Characteristic	Univariable				Multivariable		
	**N**	**OR**	**95% CI**	**p-value**	**aOR**	**95% CI**	**p-value**
**Age group (sexual partner)**	2,906						
*15-24*		—	—		—	—	
*25-34*		1.30	0.99, 1.70	0.056	1.10	0.70, 1.71	0.7
*35-49*		1.33	1.03, 1.73	**0.030**	0.88	0.46, 1.68	0.7
*50+*		1.60	1.18, 2.16	**0.003**	0.91	0.33, 2.50	0.8
**Age group (index)**	2,906						
*15-24*		—	—		—	—	
*25-34*		0.91	0.66, 1.25	0.55	1.03	0.65, 1.62	>0.9
*35-49*		1.13	0.84, 1.53	0.42	1.85	0.95, 3.57	0.069
*50+*		1.40	1.00, 1.95	**0.050**	3.84	1.35, 10.9	**0.011**
**Enrolled in HIV care**	2,906						
*Unknown*		—	—		—	—	
*enrolled*		0.71	0.52, 0.97	**0.031**	0.77	0.52, 1.14	0.2
**Occupation (index)**	2,906						
*Formal Employment*		—	—		—	—	
*Not employed*		1.26	0.89, 1.81	0.20	1.61	1.01, 2.57	**0.043**
*Self employed*		1.39	1.05, 1.86	**0.024**	1.75	1.20, 2.54	**0.003**
*Informal Employment*		1.18	0.84, 1.65	0.35	1.38	0.87, 2.17	0.2
**Marital status (index)**	2,906						
*Married Monogamy*		—	—		—	—	
*Widowed*		0.74	0.74, 0.75	**<0.001**	0.65	0.38, 1.12	0.12
*Separated/ Divorced*		0.81	0.81, 0.82	**<0.001**	0.79	0.56, 1.13	0.2
*Married Polygamy*		1.18	1.18, 1.19	**<0.001**	1.37	0.72, 2.61	0.3
*Single*		0.59	0.59, 0.59	**<0.001**	0.50	0.37, 0.69	**<0.001**
**Client entry point (Index)**	2,906						
*Community Testing*		—	—		—	—	
*PITC Hospital Patient Testing*		3.00	1.42, 6.34	**0.004**	2.11	0.77, 5.74	0.14
*Index testing*		4.25	1.99, 9.07	**<0.001**	2.35	0.86, 6.45	0.10
*Social Network strategy*		3.30	1.48, 7.35	**0.003**	2.83	1.02, 7.87	**0.047**
*PITC non-patients*		2.55	1.13, 5.76	**0.024**	1.87	0.64, 5.43	0.2
**Population type (index)**	2,906						
*General Population*		—	—		—	—	
Population at elevated risk		0.66	0.49, 0.88	**0.005**	1.00	0.60, 1.67	>0.9
**Index client type (index)**	2,906						
*Secondary*		—	—		—	—	
*Primary*		0.71	0.71, 0.72	**<0.001**	0.75	0.57, 0.99	**0.041**
**PS approach (sexual partner)**	2,906						
*Contract Referral*		—	—		—	—	
*Passive Referral*		1.32	0.88, 1.98	0.18	1.56	0.93, 2.63	0.092
*Dual Referral*		1.92	1.30, 2.82	**0.001**	2.22	1.34, 3.66	**0.002**
*Provider Referral*		1.66	1.18, 2.34	**0.004**	1.76	1.13, 2.76	**0.013**
**Occupation (sexual partner)**	2,906						
*Not employed*		—	—		—	—	
*Self employed*		1.25	1.00, 1.56	**0.048**	0.93	0.69, 1.27	0.7
*Informal Employment*		1.38	1.04, 1.81	**0.023**	1.39	0.94, 2.06	0.10
*Formal Employment*		1.74	1.28, 2.37	**<0.001**	1.55	1.04, 2.33	**0.033**
**Knowledge HIV status (sexual partner)**	2,906						
*Negative*		—	—		—	—	
*Unknown*		1.32	1.06, 1.65	**0.014**	0.84	0.62, 1.13	0.3
**HIV test outcome (sexual partner)**	2,906						
*Negative*		—	—		—	—	
*Positive*		3.84	3.01, 4.89	**<0.001**	4.70	3.39, 6.52	**<0.001**

Abbreviations: CI = Confidence Interval, OR = Odds Ratio, aOR = adjusted Odds Ratio, HIV = human immunodeficiency virus, PS = partner services, PITC = Provider-initiated testing and counseling.

a. Dependent variable: Duration from elicitation to receiving HIV test: timely testing is within 14 days.

b. Reference variable: indicated as.

c. Variables adjusted for: Index client age, occupation, marital status, HIV care enrollment status, Client entry point, index client type, population type, partner services approach, sexual partner age, occupation, and prior knowledge of HIV status.

Compared with sexual partners whose HIV status was unknown, sexual partners who already enrolled in HIV care had lower odds of timely HIV testing in the adjusted analysis (OR=0.71; 95% CI: 0.52-.97); however, this association was insignificant in the adjusted model.

Regarding index clients characteristics, sexual partners of self-employed index clients had 1.75 times higher odds of timely testing compared with sexual partners of formerly employed index clients (aOR: 1.75; 95% CI: 1.20-2.54). Likewise, sexual partners of unemployed index clients had higher odds of timely HIV testing than sexual partners of formerly employed index clients in the unadjusted analysis (OR: 1.61; 95% CI: 1.01-2.57), although this association was not retained in the adjusted model.

Compared with sexual partners of index clients in a monogamous marriages, sexual partners of single index clients had lower odds of timely HIV testing(OR: 0.5; 95% CI: 0.37-0.69); however, marital status was not significantly associated with timely HIV testing after adjustment. Sexual partners of index clients identified through the social network strategy had significantly higher odds of timely testing than sexual partners of index clients identified through community testing (aOR: 2.83; 95% CI: 1.02-7.87).

Notification approach was also significantly associated with timely testing. Compared with sexual partners of index clients who preferred dual referral, sexual partners of index clients preferring contractual referral had more than twice the odds of timely HIV testing (aOR=2.22; 95% CI: 1.34-3.66). Furthermore, compared with sexual partners of index clients who chose contract referral, sexual partners of index clients that opted for provider referral had 1.76 times higher odds of timely HIV testing (aOR=1.76; 95% CI: 1.13-2.76).

Compared with sexual partners who eventually tested HIV negative, those with an unknown HIV status had higher odds of timely HIV testing in the unadjusted analysis (OR=1.32; 95% CI: 1.06-1.65); however, this association was not statistically significant after adjustment. In contrast, sexual partners who ultimately tested HIV positive had substantially higher odds of timely HIV testing than those who tested HIV-negative (aOR= 4.70; 95% CI: 3.39-6.52).

## Discussion

This study aimed to determine factors associated with timely HIV testing of partner s identified through PS in Lower eastern Kenya. The findings showed that the dual and provider referral approaches was significantly associated with HIV testing within 14 days of elicitation. These findings reinforce the role of active, provider-supported notification approaches in enhancing early case identification. The dual referral was found to be the best approach, taking 14 days or less to test most of the sexual partners for HIV. This aligns with evidence from Côte d’Ivoire and Central Kenya, which demonstrated increased acceptability and effectiveness when providers actively engage in the disclosure and testing process [7–9]. The possible explanation for this would be that in provider approach, the burden of notifying the sexual partners on the risk and need for HIV testing is taken off the index client and the client has no role; hence index clients would prefer it over any other notification approach. Notably, our findings contrast with results from Malawi and Tanzania, where passive referrals were more commonly preferred [10,11]. These differences may reflect contextual variations in sociocultural norms, health system infrastructure, and partner services policy implementation fidelity. In Kenya, national guidelines increasingly emphasize assisted notification, which may be contributing to both provider and client preference for more direct methods.

The age of index clients was significantly associated with a shorter testing duration with sexual partners of index clients aged above 50 years more likely to test within 14 days. This is consistent with prior findings suggesting that older clients may have more stable relationships, greater health-seeking behaviors, or higher perceived risk awareness, all of which can facilitate quicker partner testing [12]. Conversely, being single compared to married monogamous was associated with delays in partner testing, likely reflecting challenges in locating or engaging past or non-regular partners. A past study carried out in Kenya showed that shorter testing duration was associated with non-spouse sexual partners [13] while another carried out in Tanzania showed a short a short testing duration among contacts of the married index clients [14].

Socioeconomic characteristics also played a role in time to HIV testing. Self-employed and unemployed index clients were more likely to have their sexual partners tested through partner services within 14 days compared to those in formal employment. This may reflect greater flexibility in scheduling or stronger rapport with sexual contacts. Similarly, sexual partners who were formally employed were more likely to be tested promptly, which may point to higher health literacy or greater trust in the healthcare system among formally employed individuals. Our findings contradict a similar study in Siaya, Kenya which found that respondents who were employed were 40% more likely not to participate in partner services as compared to those who were self-employed (OR=1.40; 95% CI = 1.89-1.97) [15].

Interestingly, sexual partners who ultimately tested HIV positive were significantly more likely to do so within 14 days. This could suggest that individuals with perceived or known exposure may seek testing more urgently, possibly due to symptoms, risk awareness, or prior discussions with the index partner. This pattern has been noted in studies evaluating the behavioral dynamics of HIV disclosure and self-assessed risk [16].

Another notable finding is the association between the Social Network Strategy (SNS) and more rapid partner testing. SNS, which involves peer-driven recruitment, may reach higher-risk individuals within close networks, thereby accelerating testing uptake due to greater trust and perceived urgency [17]]. Similarly, primary index clients (i.e., newly diagnosed) were less likely to have their sexual partners tested promptly compared to secondary index clients (diagnosed as a sexual partner of a primary index client), possibly reflecting initial hesitation in disclosure or less familiarity with the health system. Secondary index clients may have been previously sensitized or engaged in care, making partner engagement easier.

These findings point to several programmatic implications; 1) A cost effective analysis would be useful to affirm whether it would be important to prioritize dual and provider referral approaches in settings where rapid HIV case identification is needed. These approaches may require more provider time but yield significantly faster results, 2) Tailoring PS Approaches based on client demographics. Additional support may be needed for younger, single, or formally employed index clients who experience more barriers to timely partner testing, 3) Scale-up of social network strategies to target high risk index clients who like secondary index are better sensitized on engaging their sexual partners, 4) Leveraging socioeconomic profiles through understanding the work patterns and behavioral dynamics of both index clients and their partners to inform contact tracing strategies, especially in urban and semi-urban areas with diverse employment profiles.

The strength of this study lies in the use of a large real-world programmatic dataset from 115 health facilities across three counties in Kenya, enhancing generalizability of the findings to similar HIV service delivery settings. Additionally, the use of generalized linear mixed models allowed for robust adjustment for potential confounders while accounting for clustering at the index client level. However, several limitations should be considered. First, the study relied on routinely collected data from partner services registers, which contain limited information on elicited sexual partners. As a result, important sexual partner-level characteristics and contextual factors that may influence HIV testing uptake and timeliness could not be examined. Second, missing or incomplete data may have affected the accuracy of some estimates, and routine program records are subject to potential underreporting or misclassification of partner notification outcomes. Third, behavioral and health system factors such HIV-related stigma, fear of HIV disclosure, relationship dynamics, and client experiences with health care providers were not captured. Future studies should consider mixed-methods approaches that incorporate qualitative data to explore these contextual influences and should expand data sources beyond partner services registers, including linkage with other clinical, behavioral, and programmatic datasets, to enable a more comprehensive assessment of factors affecting partner testing outcomes.

## Conclusions

Dual and provider referral approaches were significantly associated with HIV testing within 14 days among sexual partners. Index and sexual partner demographics, test modality, and employment status influenced outcomes. Future studies could focus on cost effectiveness analysis to inform the feasibility of investing in provider-supported strategies to accelerate case identification and linkage to care in Kenya and similar settings.

## Supporting information

S1 Data The Partner Services paper data is meant to be published alongside the manuscript and for reviewers to have a look at (if need be) as they review the paper. It can be accessed as an attachment under supplementary information, in the submission.
(XLSX)
