# Supplementary material for: Factors associated with timely HIV testing of sexual partners identified through partner services in lower eastern Kenya, 2023–2024
Source: PLOS Glob Public Health. 2026 Nov 13;6(11):e0007171. doi: 10.1371/journal.pgph.0007171 (PMC13766222; doi:10.1371/journal.pgph.0007171)
Supplement: Supplementary file 1 [file pgph.0007171.s002.docx]

**PLOS Global Public Health-** **PGPH-D-26-01441 Review**

**Reviewer comments**

This is a well written paper and provides a great addition to literature around optimizing partner services by focusing on timely HIV testing of sexual and injecting partners of people living with HIV. It will be a good and novel contribution to the field.

**Abstract**

The abstract would benefit from more clarity of terms. In the methods, please define index clients.

The main outcome of this paper is timeliness. Please define how you measured timeliness in the methods section.

In the results section, the authors are missing some details on timing and overall study population. Please include date when this study was conducted. In addition to proportions, include the number of index and sexual partners included in the study and from how many facilities.

**Overall**

Overall, the authors use terms such as HIV infected or uninfected or HIV positive. HIV positive maybe ideal when referring to the test but not the individuals. Please use the more person-centered language ie person living with HIV or person without HIV or woman living with HIV etc.

**Methods**

In the study design and setting, the authors should include the number of facilities.

While the authors define primary and secondary index clients, the distinction between the two is not clear. Both options form the starting point of PS for them to be considered as index clients.

The operational definition of the primary outcome, timeliness, is not clearly specified. Was this the time of elicitation related to when the index client elicited partners? If so, please include that in the definition of outcomes. In the definition, authors should also specify that this refers to the testing of the sexual partners of index clients.

The analysis lacks details on how the authors controlled for confounding and what variables were adjusted for. Important variables especially of partner type (regular vs casual), willingness to disclosure and accessibility to services(rural vs urban) which may both influence timeliness but also the type of PS strategy that is selected by the index are missing in the paper.

The authors define partner services as identifying sexual and injecting partners of index clients. The authors seem to focus on sexual partners. It is not clear if the partners elicited included were sexual, injecting or both.

**Results**

Overall, the results would benefit from clearer specification of the comparison groups. Because the outcomes are based on sexual partner testing, analyses using index client characteristics should frame comparisons at the level of partners—for example, referring to “sexual partners of index clients” as the comparator rather than the index clients themselves.

The authors use the terms “sexual partners” and “contact clients” inconsistently throughout the results. One term should be used consistently.

The sexual partner characteristics in table 1 are limited. Did the authors collect any data on marital status and risk behaviors of the partners or population type?

The results state that sexual partners had a preferred HTS notification approach. This is also indicated in table 1. It is not clear if the HTS notification strategy is what was chosen by the index or the strategy that was finally used to reach the partners.

The results mention “adjusting for other factors in the model”. These factors are not specified in the table footnotes, methods or results narrative.

In line 179, the authors mention index clients with unknown HIV status. In the definition of PS, the index clients are people living with HIV. This needs to be clarified.
