# Supplementary material for: Factors associated with timely HIV testing of sexual partners identified through partner services in lower eastern Kenya, 2023–2024
Source: PLOS Glob Public Health. 2026 Nov 13;6(11):e0007171. doi: 10.1371/journal.pgph.0007171 (PMC13766222; doi:10.1371/journal.pgph.0007171)
Supplement: Supplementary file 2 [file pgph.0007171.s004.docx]

| **Reviewers’ and Editor’s Comment** | **Response** | **Changes Made** |
| --- | --- | --- |
| This is a well-written paper and provides a great addition to literature around optimizing partner services by focusing on timely HIV testing of sexual and injecting partners of people living with HIV. It will be a good and novel contribution to the field. | Thank you | None |
| The abstract would benefit from more clarity of terms. In the methods, please define index clients. | Index client has been defined in both the abstract and main article  We have also scanned through to enhance clarity of the terms used | **Lines 27-28** “…from index clients (PLHIV who consented to partner services and provided information on sexual partners for notification and testing)”, and  **Line 102** “The study population was PLHIV who had consented to receive partner services (index clients)” |
| The main outcome of this paper is timeliness. Please define how you measured timeliness in the methods section. | We have updated the methods section of the abstract and manuscript to include a definition of timeliness | **Line 30 -31** “Factors associated with timely partner testing, defined as HIV testing within 14 days of elicitation” and  **Lines 132 – 134** “…considering timeliness as sexual partners being tested within 14 days” |
| In the results section, the authors are missing some details on timing and overall study population. Please include date when this study was conducted. In addition to proportions, include the number of index and sexual partners included in the study and from how many facilities. | We have updated the methods section by including the number of facilities included in the study and the timing of the study.  We have also updated the results section with the overall study population size, and number for stated results proportions | **Line 28 – 29** “Index clients…….who participated in partner services between January 2023 and December 2024 across 115 health facilities. Sexual partners elicited from these index clients were included if they had received HIV test by 30 June 2025”  **Line 170-171**  **Lines 33 – 35** “A total of 2210 index clients and 2906 sexual partners were included in the study. Among the index clients, majority were females 63% (n=1393), while most of the sexual partners were males at 58% (n=1679). Provider referral was the most preferred approach at 63% (n=1831), while contract referral was the least preferred at 8% (n=273)” |
| The sexual partner characteristics in table 1are limited. Did the authors collect any data on marital status and risk behaviors of the partners or population type | Unfortunately, the sexual partner characteristics collected were limited to data available in the Partner Service Registers. We have included this as a limitation of this study, recommending mixed methods approach in future studies, and where feasible expansion of data source beyond the partner service records. | **Line 286 – 297 reads** “However, several limitations should be considered. First, the study relied on routinely collected data from partner services registers, which contain limited information on elicited sexual partners. As a result, important sexual partner-level characteristics and contextual factors that may influence HIV testing uptake and timeliness could not be examined. Further, missing or incomplete data may have affected the accuracy of some estimates, and routine program records are subject to potential underreporting or misclassification of partner notification outcomes. The behavioral and health system factors such HIV-related stigma, fear of HIV disclosure, relationship dynamics, and client experiences with health care providers were not equally captured. Future studies should consider mixed methods approaches that incorporate qualitative data to explore these contextual influences and should expand data sources beyond partner services registers, including linkage with other clinical, behavioral, and programmatic datasets, to enable a more comprehensive assessment of factors affecting partner testing outcomes |
| Overall, the authors use terms such as HIV infected or uninfected or HIV positive. HIV positive may be ideal when referring to the test but not the individuals. Please use the more person-centered language i.e. person living with HIV or person without HIV or woman living with HIV etc | Person-centered terminologies have been corrected/used, for example as shown in lines 73, 74, 92, 94 | For example, page 4 line 75, now reads ‘...people living with HIV’. |
| **Methods**  In the study design and setting, the authors should include the number of facilities. | We have added the number of facilities. | **Line 121** now reads “..... from 115 Public health facilities under the Ministry of Health (MOH)” |
| While the authors define primary and secondary index clients, the distinction between the two is not clear. Both options form the starting point of PS for them to be considered as index clients. | This has been clarified to show a distinction between primary and secondary index clients | **Line 108 - 111** “ The index clients were either primary (i.e. initial person who tests positive for HIV then provides names of sexual partners they wish to be traced for HIV testing as well. ) or secondary (i.e. sexual partners of the primary index client who were traced, tested and subsequently diagnosed with HIV” |
| The operational definition of the primary outcome, timeliness, is not clearly specified. Was this the time of elicitation related to when the index client elicited partners? If so, please include that in the definition of outcomes. In the definition, authors should also specify that this refers to the testing of the sexual partners of index clients. | Timeliness as the primary outcome has been updated to time taken in days from elicitation of sexual partners to the time they were tested for HIV. | **Line 132 – 134** “The primary outcome for this study was days between elicitation of sexual partners and their HIV testing. Consistent with the national guideline recommendation, HIV testing conducted within 14 days of partner elicitation was classified as timely” |
| The analysis lacks details on how the authors controlled for confounding and what variables were adjusted for. Important variables especially of partner type (regular vs casual), willingness to disclosure and accessibility to services (rural vs urban) which may both influence timeliness but also the type of PS strategy that is selected by the index are missing in the paper. | We have provided details on how we controlled for confounders. We have also noted our limitations on missing potential confounders that limited our analysis. | **Line 157 – 160** “Variables adjusted for included index client age, occupation, marital status, HIV care enrollment status, Client entry point, index client type, population type, partner notification approach, sexual partner age, occupation, and prior knowledge of HIV status. These variables were included simultaneously to minimize potential confounding” |
| The results mention, “adjusting for other factors in the model”. These factors are not specified in the table footnotes, methods or results narrative | Factors have been specified in the narrative and included in table 2 as a footnote |  |
| The authors define partner services as identifying sexual and injecting partners of index clients. The authors seem to focus on sexual partners. It is not clear if the partners elicited included were sexual, injecting or both. | We acknowledge that Partner services includes both sexual and injecting drug partners. However, this study included sexual partners only; no injecting drug contacts were included. This exclusion has been included in the study population section. | **Line 104 – 107** “According to the national PS guidelines, eligible partners include both sexual and injecting drug partners. However, this study was limited to sexual partners excluding drug-injecting partners due to their very low numbers and the study's focus on sexual partner notification outcomes.” |
| Results  Overall, the results would benefit from clearer specification of the comparison groups. Because the outcomes are based on sexual partner testing, analyses using index client characteristics should frame comparisons at the level of partners—for example, referring to “sexual partners of index clients” as the comparator rather than the index clients themselves. | We have re-written the results consistently using **"sexual partners of index clients"** as the comparison group whenever the explanatory variable is an index-client characteristic, thereby aligning the narrative with the outcome being measured among sexual partners. | **Line 190 - 228** |
| The authors use the terms “sexual partners” and “contact clients” inconsistently throughout the results. One term should be used consistently. | We have corrected consistently using sexual partners across the manuscript. Including the title |  |
| The results state that sexual partners had a preferred HTS notification approach. This is also indicated in table 1. It is not clear if the HTS notification strategy is what was chosen by the index or the strategy that was finally used to reach the partners. | In partner services, the notification approach used is chosen by the index client, from the available options in use to notify sexual partners of the need to test for HIV. During the counselling session, the health care workers (HCW) explain to the index client options available for notifying their sexual partner(s) about their risk and the need to test for HIV. The index client makes an informed choice on how they wish their sexual partners to be reached. We have included this description of the intervention in the introduction section. | **Line 71 - 73** |
| In line 179, the authors mention index clients with unknown HIV status. In the definition of PS, the index clients are people living with HIV. This needs to be clarified. | This was a typing error. We meant to write sexual partners, not contact clients as initially captured. We have changed to sexual partners. |  |
| **Journal Requirements:**    1.  Please note that PLOS Global Public Health has specific guidelines on code sharing for submissions in which author-generated code underpins the findings in the manuscript. In these cases, all author-generated code must be made available without restrictions upon publication of the work. Please review our guidelines at https://journals.plos.org/globalpublichealth/s/materials-and-software-sharing#loc-sharing-code and ensure that your code is shared in a way that follows best practice and facilitates reproducibility and reuse. | We take note of these requirements and are ready to comply fully. |  |
| 2. Please amend your detailed Financial Disclosure statement. This is published with the article. It must therefore be completed in full sentences and contain the exact wording you wish to be published. |  |  |
| i. Please clarify all sources of financial support for your study. List the grants, grant numbers, and organizations that funded your study, including funding received from your institution. Please note that suppliers of material support, including research materials, should be recognized in the Acknowledgements section rather than in the Financial Disclosure. | This implementation project was supported by the U.S. Centers for Disease Control and ‎Prevention ‎‎(CDC) with funding from the President's Emergency Plan for AIDS Relief (PEPFAR) ‎under the ‎terms of: GH21-2116 :Achieving and Sustaining Epidemic Control Through ‎Comprehensive ‎HIV/AIDS Prevention, Care and Treatment Services in Machakos, Makueni and ‎Kitui Counties in ‎Kenya (NU2GGH002333)‎ | Line 325 - 330 |
| ii. State the initials, alongside each funding source, of each author to receive each grant. For example: "This work was supported by the National Institutes of Health (####### to AM; ###### to CJ) and the National Science Foundation (###### to AM)." | This work was supported by This implementation ‎project was supported ‎by the U.S. Centers for ‎Disease Control and ‎‎Prevention ‎‎(CDC) with ‎funding from the ‎President's Emergency ‎Plan for AIDS Relief ‎‎(PEPFAR: # GH21-2116 and #NU2GGH002333)‎ to EK |  |
| iii. State what role the funders took in the study. If the funders had no role in your study, please state: “The funders had no role in study design, data collection and analysis, decision to publish, or preparation of the manuscript.”  iv. If any authors received a salary from any of your funders, please state which authors and which funders. | The funders had no role in study design, data collection and ‎analysis, decision to publish, or preparation of the ‎manuscript.‎ | Line 329 - 330 |
| 3. In the online submission form, you indicated that “The data used in this study are available from the corresponding author upon reasonable request and subject to ethical approvals.”.  All PLOS journals now require all data underlying the findings described in their manuscript to be freely available to other researchers, either   1. In a public repository,   2. Within the manuscript itself, or   3. Uploaded as supplementary information.  This policy applies to all data except where public deposition would breach compliance with the protocol approved by your research ethics board. If your data cannot be made publicly available for ethical or legal reasons (e.g., public availability would compromise patient privacy), please explain your reasons by return email and your exemption request will be escalated to the editor for approval. Your exemption request will be handled independently and will not hold up the peer review process, but will need to be resolved should your manuscript be accepted for publication. One of the Editorial team will then be in touch if there are any issues. | Owing to the fact that the data upon which these manuscript is based has identifiers such as the facility names, the names of index clients and names of sexual contacts as well as the HIV status, we have made reasonable efforts to de-identify this data and including the aggregate level data within this manuscript itself in conformity with PLOS policy requirements. | Line 336 - 340 |
| 4. Please ensure that your Ethics Statement is available in its entirety at the beginning of your Methods section, under a subheading 'Ethics Statement'. | The paper is based on an approved evaluation protocol by the AMREF Ethics and Scientific ‎Review Committee under the approval number AMREF-ESRC P1421/23 and the University of ‎Maryland, Baltimore Institutional Review Board under approval number HP-00106405. The ‎assessment protocol was also reviewed as per the U.S. CDC human research protection ‎procedures and was determined to be non-research.‎ | Lines:**93 - 100** |
| 5. We note that you have included your Figures within the body of your manuscript. Please remove the Figures from the body of your manuscript and upload them as separate Figure files in .tif or .eps format.    For more information about figure files please see our guidelines:  <https://journals.plos.org/globalpublichealth/s/figures>  <https://journals.plos.org/globalpublichealth/s/figures#loc-file-requirements>  If the reviewer comments include a recommendation to cite specific previously published works, please review and evaluate these publications to determine whether they are relevant and should be cited. There is no requirement to cite these works unless the editor has indicated otherwise. | Figure 1 has been removed from the manuscript body and uploaded separately |  |
| **Please review your reference** list to ensure that it is complete and correct. If you have cited papers that have been retracted, please include the rationale for doing so in the manuscript text, or remove these references and replace them with relevant current references. Any changes to the reference list should be mentioned in the rebuttal letter that accompanies your revised manuscript. If you need to cite a retracted article, indicate the article’s retracted status in the References list and also include a citation and full reference for the retraction notice. | Reference list checked and is complete and correct. | No changes made to the manuscript in regard to this. |
| **Editor Comments**  Recommend minor revision before acceptance. Please consider the following issues during revision:   1) Improve clarity in the abstract by defining “Index client” in the methods section and clearly describing how the primary outcome, timeliness, was measured | These changes have been effected in the revised abstract |  |
| 2) Include additional details in the abstract results section, specifically  - The study period - The number of index clients included - The number of sexual partners included in the analysis - The number of facilities | The study period, number of index clients, number of sexual partners and number of facilities updated in the abstract results section | Page 2 lines 28, 29 and 30 |
| 3) Please verify the terminology used in line 32 and confirm whether “contract referral” should instead be “Contact referral | The terminology used is contract referral where a HCW enters into a contract with the index client to notify sexual partners of the need to test | Line 68 |
| 4) Consider adopting person-centered HIV terminology throughout the manuscript. Terms such as “HIV infected”,”uninfected”, or references to individuals as “HIV positive” should be replaced with more person-centered alternatives such as”people living with HIV”, where appropriate | Terminology used consistently throughout the paper | Example Lines 75, 76, 102 |
| 5) In the methods section, please provide:  - The number of participating facilities, - A clearer distinction between primary and secondary index clients, - An explicit operational definition of timeliness and how it was measured, and - Details of the variables included in adjusted analyses and approaches used to control for confounding. | Number of facilities captured in the method section  A clearer distinction between primary and secondary index clients has been updated  Operational definition of timeliness updated  Confounding factors included in the methods section | Page 5 Line 121  Page 4 lines 108 to 112  Page 5 line 132  Page 6 line 148-160 |
| 6) Clarify whether the study included sexual partners, injecting partners, or both, since the definition of partner services includes both groups while the analysis appears to focus primarily on sexual partners. | We restricted inclusion for sexual partners |  |
| 7) Improve consistency in terminology throughout the results section, particularly regarding the use of "sexual partners" and "contact clients"   The sexual partner characteristics in table 1 are limited. Did the authors collect any data on marital status and risk behaviors of the partners or population type? | Effected across the manuscript. We have used sexual partner in place of contact clients  Marital status and population type was collected as reported by the index clients. Risk behaviours of sexual partners was not collected | Table 1 |
| 8) Clarify whether the reported HTS notification strategy reflects the option selected by the index client or the strategy ultimately used to reach partners.   The results state that sexual partners had a preferred approach. This is also indicated in table 1. It is not clear if the HTS notification strategy is what was chosen by the index or the strategy that was finally used to reach the partners | The notification approach is selected by the index client upon guidance by the health care worker to select the most appropriate approach, from the available options, to be used to notify their sexual partners of the need to test for HIV. |  |
| 9) Clarify references to index clients with unknown HIV status, particularly given the definition of index clients as people living with HIV.   In line 179, the authors mention index clients with unknown HIV status. In the definition of PS, the index clients are People living with HIV. This needs to be clarified | This was a typo meant to be sexual partners, not contact clients. This has been rectified |  |
