# Supplementary material for: Factors associated with timely HIV testing of sexual partners identified through partner services in lower eastern Kenya, 2023–2024
Source: PLOS Glob Public Health. 2026 Nov 13;6(11):e0007171. doi: 10.1371/journal.pgph.0007171 (PMC13766222; doi:10.1371/journal.pgph.0007171)
Supplement: Peer review history

# Peer review: Decision Letter 0

**DOI:** 10.1371/journal.pgph.0007171.r001

**Authors:** Mavis Pearl Kwabla

6 Jul 2026

PGPH-D-26-01441

Factors associated with timely HIV testing of contacts identified through Partner Services in lower eastern Kenya, 2023-2024

PLOS Global Public Health

Dear Dr. Mutiso Jones,

Thank you for submitting your manuscript to PLOS Global Public Health. After careful consideration, we feel that it has merit but does not fully meet PLOS Global Public Health’s publication criteria as it currently stands. Therefore, we invite you to submit a revised version of the manuscript that addresses the points raised during the review process.

Further details can be found in the reviewers' comments as well as the comments to the authors.

Please submit your revised manuscript by July 17th, 2026. If you will need more time than this to complete your revisions, please reply to this message or contact the journal office at globalpubhealth@plos.org. When you're ready to submit your revision, log on to https://www.editorialmanager.com/pgph/ and select the 'Submissions Needing Revision' folder to locate your manuscript file.

Please include the following items when submitting your revised manuscript:

- A letter that responds to each point raised by the editor and reviewer(s). You should upload this letter as a separate file labeled 'Response to Reviewers'.
- A marked-up copy of your manuscript that highlights changes made to the original version. You should upload this as a separate file labeled 'Revised Manuscript with Track Changes'.
- An unmarked version of your revised paper without tracked changes. You should upload this as a separate file labeled 'Manuscript'.

Guidelines for resubmitting your figure files are available below the reviewer comments at the end of this letter.

As the corresponding author, your ORCID iD is verified in the submission system and will appear in the published article. PLOS supports the use of ORCID, and we encourage all coauthors to register for an ORCID iD and use it as well. Please encourage your coauthors to verify their ORCID iD within the submission system before final acceptance, as unverified ORCID iDs will not appear in the published article. *Only*  the individual author can complete the verification step; PLOS staff *cannot*  verify ORCID iDs on behalf of authors.

We look forward to receiving your revised manuscript.

Kind regards,

Mavis Pearl Kwabla, Master of Public Health

Academic Editor

PLOS Global Public Health

**Journal Requirements:**

1.  Please note that PLOS Global Public Health has specific guidelines on code sharing for submissions in which author-generated code underpins the findings in the manuscript. In these cases, all author-generated code must be made available without restrictions upon publication of the work. Please review our guidelines at https://journals.plos.org/globalpublichealth/s/materials-and-software-sharing#loc-sharing-code and ensure that your code is shared in a way that follows best practice and facilitates reproducibility and reuse.

2. Please amend your detailed Financial Disclosure statement. This is published with the article. It must therefore be completed in full sentences and contain the exact wording you wish to be published.

i. Please clarify all sources of financial support for your study. List the grants, grant numbers, and organizations that funded your study, including funding received from your institution. Please note that suppliers of material support, including research materials, should be recognized in the Acknowledgements section rather than in the Financial Disclosure.

ii. State the initials, alongside each funding source, of each author to receive each grant. For example: "This work was supported by the National Institutes of Health (####### to AM; ###### to CJ) and the National Science Foundation (###### to AM)."

iii. State what role the funders took in the study. If the funders had no role in your study, please state: “The funders had no role in study design, data collection and analysis, decision to publish, or preparation of the manuscript.”

iv. If any authors received a salary from any of your funders, please state which authors and which funders.

3. In the online submission form, you indicated that “The data used in this study are available from the corresponding author upon reasonable request and subject to ethical approvals.”.

All PLOS journals now require all data underlying the findings described in their manuscript to be freely available to other researchers, either

1. In a public repository,

2. Within the manuscript itself, or

3. Uploaded as supplementary information.

This policy applies to all data except where public deposition would breach compliance with the protocol approved by your research ethics board. If your data cannot be made publicly available for ethical or legal reasons (e.g., public availability would compromise patient privacy), please explain your reasons by return email and your exemption request will be escalated to the editor for approval. Your exemption request will be handled independently and will not hold up the peer review process, but will need to be resolved should your manuscript be accepted for publication. One of the Editorial team will then be in touch if there are any issues.

4. Please ensure that your Ethics Statement is available in its entirety at the beginning of your Methods section, under a subheading 'Ethics Statement'.

5. We note that you have included your Figures within the body of your manuscript. Please remove the Figures from the body of your manuscript and upload them as separate Figure files in .tif or .eps format.

For more information about figure files please see our guidelines:

https://journals.plos.org/globalpublichealth/s/figures

https://journals.plos.org/globalpublichealth/s/figures#loc-file-requirements

If the reviewer comments include a recommendation to cite specific previously published works, please review and evaluate these publications to determine whether they are relevant and should be cited. There is no requirement to cite these works unless the editor has indicated otherwise.

Please review your reference list to ensure that it is complete and correct. If you have cited papers that have been retracted, please include the rationale for doing so in the manuscript text, or remove these references and replace them with relevant current references. Any changes to the reference list should be mentioned in the rebuttal letter that accompanies your revised manuscript. If you need to cite a retracted article, indicate the article’s retracted status in the References list and also include a citation and full reference for the retraction notice.

**Additional Editor Comments (if provided):**

Dear Authors

Thank you for the opportunity to review this interesting and relevant manuscript. The study addresses an important aspect of HIV partner services by examining the timeliness of HIV testing among partners of people living with HIV, and it represents a useful contribution to the existing literature.

After reviewing the manuscript and considering the comments from both reviewers, I recommend minor revision before acceptance. Please consider the following issues during revision:

1. Improve clarity in the abstract by defining "index clients" in the methods section and clearly describing how the primary outcome, timeliness, was measured.

2. Include additional details in the abstract results section, specifically:

o the study period,

o the number of index clients included,

o the number of sexual partners included in the analysis, and

o the number of participating facilities.

3. Please verify the terminology used in Line 32 and confirm whether "contract referral" should instead be "contact referral".

4. Consider adopting person-centred HIV terminology throughout the manuscript. Terms such as "HIV infected", "uninfected", or references to individuals as "HIV positive" should be replaced with more person-centred alternatives such as "people living with HIV" or "people without HIV", where appropriate.

5. In the methods section, please provide:

o the number of participating facilities,

o a clearer distinction between primary and secondary index clients,

o an explicit operational definition of timeliness and how it was measured, and

o details of the variables included in adjusted analyses and approaches used to control for confounding.

6. Clarify whether the study included sexual partners, injecting partners, or both, since the definition of partner services includes both groups while the analysis appears to focus primarily on sexual partners.

7. Improve consistency in terminology throughout the results section, particularly regarding the use of "sexual partners" and "contact clients".

8. Clarify whether the reported HTS notification strategy reflects the option selected by the index client or the strategy ultimately used to reach partners.

9. Clarify references to index clients with unknown HIV status, particularly given the definition of index clients as people living with HIV.

The manuscript is well written and addresses an important implementation question. Addressing the above comments and those provided by Reviewer 1 will strengthen the clarity and interpretability of the findings.

Kind regards,

Dr. Mavis Pearl Kwabla (PhD, MPH)

Academic Editor

[Note: HTML markup is below. Please do not edit.]

Reviewers' comments:

Reviewer's Responses to Questions

**Comments to the Author**

1. Does this manuscript meet PLOS Global Public Health’s publication criteria?

Reviewer #1: Yes

**********

2. Has the statistical analysis been performed appropriately and rigorously?

Reviewer #1: No

**********

3. Have the authors made all data underlying the findings in their manuscript fully available (please refer to the Data Availability Statement at the start of the manuscript PDF file)?

The PLOS Data policy

Reviewer #1: Yes

**********

4. Is the manuscript presented in an intelligible fashion and written in standard English?

Reviewer #1: Yes

**********

**Reviewer #1:**  PLOS Global Public Health- PGPH-D-26-01441 Review

Reviewer comments

This is a well written paper and provides a great addition to literature around optimizing partner services by focusing on timely HIV testing of sexual and injecting partners of people living with HIV. It will be a good and novel contribution to the field.

Abstract

The abstract would benefit from more clarity of terms. In the methods, please define index clients.

The main outcome of this paper is timeliness. Please define how you measured timeliness in the methods section.

In the results section, the authors are missing some details on timing and overall study population. Please include date when this study was conducted. In addition to proportions, include the number of index and sexual partners included in the study and from how many facilities.

Overall

Overall, the authors use terms such as HIV infected or uninfected or HIV positive. HIV positive maybe ideal when referring to the test but not the individuals. Please use the more person-centered language ie person living with HIV or person without HIV or woman living with HIV etc.

Methods

In the study design and setting, the authors should include the number of facilities.

While the authors define primary and secondary index clients, the distinction between the two is not clear. Both options form the starting point of PS for them to be considered as index clients.

The operational definition of the primary outcome, timeliness, is not clearly specified. Was this the time of elicitation related to when the index client elicited partners? If so, please include that in the definition of outcomes. In the definition, authors should also specify that this refers to the testing of the sexual partners of index clients.

The analysis lacks details on how the authors controlled for confounding and what variables were adjusted for. Important variables especially of partner type (regular vs casual), willingness to disclosure and accessibility to services(rural vs urban) which may both influence timeliness but also the type of PS strategy that is selected by the index are missing in the paper.

The authors define partner services as identifying sexual and injecting partners of index clients. The authors seem to focus on sexual partners. It is not clear if the partners elicited included were sexual, injecting or both.

Results

Overall, the results would benefit from clearer specification of the comparison groups. Because the outcomes are based on sexual partner testing, analyses using index client characteristics should frame comparisons at the level of partners—for example, referring to “sexual partners of index clients” as the comparator rather than the index clients themselves.

The authors use the terms “sexual partners” and “contact clients” inconsistently throughout the results. One term should be used consistently.

The sexual partner characteristics in table 1 are limited. Did the authors collect any data on marital status and risk behaviors of the partners or population type?

The results state that sexual partners had a preferred HTS notification approach. This is also indicated in table 1. It is not clear if the HTS notification strategy is what was chosen by the index or the strategy that was finally used to reach the partners.

The results mention “adjusting for other factors in the model”. These factors are not specified in the table footnotes, methods or results narrative.

In line 179, the authors mention index clients with unknown HIV status. In the definition of PS, the index clients are people living with HIV. This needs to be clarified.

**********

what does this mean?). If published, this will include your full peer review and any attached files.

**Do you want your identity to be public for this peer review?**  If you choose “no”, your identity will remain anonymous but your review may still be made public.

For information about this choice, including consent withdrawal, please see our Privacy Policy

Reviewer #1: No

**********

[NOTE: If reviewer comments were submitted as an attachment file, they will be attached to this email and accessible via the submission site. Please log into your account, locate the manuscript record, and check for the action link "View Attachments". If this link does not appear, there are no attachment files.]

**Figure Resubmissions:**

Attachment Submitted filename: PLOS Global Public Health- PGPH-D-26-01441 Review 5.14.26.docx

# Author comment: Author response to Decision Letter 1

**DOI:** 10.1371/journal.pgph.0007171.r002

11 Sep 2026

Attachment Submitted filename: Response to Reviewers.docx

# Editor report: Decision Letter 1

**DOI:** 10.1371/journal.pgph.0007171.r003

**Authors:** Mavis Pearl Kwabla

18 Oct 2026

Factors associated with timely HIV testing of sexual partners identified through Partner Services in lower eastern Kenya, 2023-2024

PGPH-D-26-01441R1

Dear Dr. Jones Mutiso,

We are pleased to inform you that your manuscript 'Factors associated with timely HIV testing of sexual partners identified through Partner Services in lower eastern Kenya, 2023-2024' has been provisionally accepted for publication in PLOS Global Public Health.

Before your manuscript can be formally accepted you will need to complete some formatting changes, which you will receive in a follow up email. A member of our team will be in touch with a set of requests.

Please note that your manuscript will not be scheduled for publication until you have made the required changes, so a swift response is appreciated.

IMPORTANT: The editorial review process is now complete. PLOS will only permit corrections to spelling, formatting or significant scientific errors from this point onwards. Requests for major changes, or any which affect the scientific understanding of your work, will cause delays to the publication date of your manuscript.

If your institution or institutions have a press office, please notify them about your upcoming paper to help maximize its impact. If they'll be preparing press materials, please inform our press team as soon as possible -- no later than 48 hours after receiving the formal acceptance. Your manuscript will remain under strict press embargo until 2 pm Eastern Time on the date of publication. For more information, please contact globalpubhealth@plos.org.

Thank you again for supporting Open Access publishing; we are looking forward to publishing your work in PLOS Global Public Health.

Best regards,

Mavis Pearl Kwabla, PhD

Academic Editor

PLOS Global Public Health

***********************************************************

Dear Authors,

Thank you for submitting the revised version of your manuscript and for addressing the comments raised during the previous round of review.

I have assessed the revised manuscript and am satisfied that you have adequately addressed the concerns raised by the reviewers. I therefore recommend the manuscript for acceptance for publication, subject to the following two minor corrections:

1. Table 2: Please change “OR” to “aOR” in the multivariable analysis column, as the multivariable logistic regression reports adjusted odds ratios.

2. Discussion section: Please replace the word “contacts” with “partners” in the first sentence to ensure consistency with the revised study objective.

Kindly incorporate these corrections into the final version of the manuscript.

Thank you for your efforts in revising the manuscript and responding to the review comments.

Best regards,

Dr Mavis Pearl Kwabla (PhD, MPH)

Academic Editor

Reviewer Comments (if any, and for reference):
